# Animal welfare and society—Part 1, The viewpoints of a philosopher

**DOI:** 10.1093/af/vfac006

**Published:** 2022-03-17

**Authors:** Gary L Francione

**Affiliations:** 1 Board of Governors Distinguished Professor of Law and Katzenbach Scholar of Law and Philosophy, Rutgers University Law School, Camden, NJ 08102, USA; 2 Visiting Professor of Philosophy, University of Lincoln, Brayford Pool, Lincoln, LN6 7TS, UK; 3 Honorary Professor of Philosophy, School of Politics, Philosophy, Language, and Communication Studies, University of East Anglia, Norwich, NR4 7TJ, UK

## What is Animal Welfare (Definition)?

We use “animal welfare” in different ways. When we are talking about animals we regard as members of our families—our “pets”—we use “animal welfare” in much the same way that we would use “welfare” when we apply it in human contexts. That is, we use “welfare” to refer to that which will facilitate the animal’s health and happiness and we apply the term in much the same way that we would if we were talking about, say, our human children; in both contexts, we seek to identify what conditions or circumstances will best ensure that they prosper and thrive.

When, however, we are talking about animals we use for food, clothing, research, testing, etc.—animals whom we use and kill for our purposes—we use “animal welfare” in a completely different way. We no longer think about welfare in terms of what will result in these animals prospering and thriving. We *cannot* think of these animals in this way because we choose to kill them in order to use them for our purposes. If we really cared about their welfare in the way that we care about the welfare of our children, dogs, and cats, etc., we would not be eating them, wearing them, or using them exclusively as resources in the various ways that we do.

“Animal welfare” in this second context seeks to identify the conditions and circumstances that will result in the efficient use of animals for our purposes. These conditions and circumstances have little to do with morality; they are primarily concerned with *economics.* Animals are *property*. They are *things* we own and that we get to value. They have no intrinsic or inherent value. They are commodities that have an economic value. Animals are unlike other things that we own because, unlike cars, cell phones, and sofas, the overwhelming portion of animals we use for food, clothing, research and testing, etc. are *sentient*; that is, they are subjectively aware. They have interests—preferences, wants, and desires. It costs money to protect their interests. And, for the most part, we spend that money to protect their interests to the extent that we get a benefit from doing so. That benefit is almost always an economic one. That is, we protect animal interests to the extent that it makes economic sense to do so; “animal welfare” is generally a matter of making sure that animal use is economically efficient.

Consider, for example, the fact that many jurisdictions have laws that require that animals—particularly large animals—be rendered unconscious before they are slaughtered. The primary reason for such a requirement is that animals who are fully conscious when they are slaughtered are likely to injure workers and incur carcass damage, which can be costly. It has been noted that animals are often not fully unconscious when slaughtered. They don’t need to be in order to be immobile—and it is mobility and not consciousness per se that poses the hazard to workers and meat quality.

Ever since the nineteenth century, we have claimed to reject the idea that animals are merely things, but we have also continued to embrace the idea that we can use and kill animals for our purposes. We invented the moral and legal fantasy of a requirement for “humane” treatment. That is, we may use and kill animals for our purposes but we are required to treat animals “humanely” and not to impose “unnecessary” suffering on them. I will discuss these ideas more below. But for present purposes, the injunction requiring “humane” treatment” and rejecting “unnecessary” suffering has generally been limited to prohibiting treatment that is *gratuitous*—that which serves no economic purpose.

“Animal welfare” is, for all intents and purposes, those practices that a rational property owner with full information would employ in the absence of any law or regulation requiring the owner to do so. The standard of “humane” treatment is often linked to what level of treatment is customary among those who use animals for the particular purpose. The reason for this is that we assume that property owners are rational and that they will not cause more damage to their animal property than is necessary to use animals for that particular purpose.

Many animal welfare measures are promoted explicitly on economic grounds. For example, Temple Grandin, the most celebrated proponent and architect of animal welfare, argues that good animal welfare increases economic benefits for producers. The campaign to adopt the controlled-atmosphere killing of chickens rather than traditional methods of chicken slaughter is based on the economic efficiency of controlled-atmosphere killing. The arguments in favor of alternatives to the farrowing crate used for pigs are also based on the economic benefits that result.

To the extent that animal welfare regulations depart from the model of economic efficiency, they don’t depart much. For example, the European Union prohibited the conventional battery cage after a 12-yr phase-in period. The EU permits “enriched cage” systems that even extremely conservative animal welfare groups criticize as not providing for significantly improved welfare benefits. Although efforts to ban cage systems in favor of “free range” and “cage free” systems are gaining traction, these measures are being phased in so as to not cause any significant economic disruption to the egg industry. Some consumers will pay more for supposedly more “humanely” produced meat and animal products and this has resulted in niche markets for these products.

## Does Animal Welfare Matter? If Yes, Why? If No, Why Not?

Animal welfare is very important; it matters a great deal—*to us*. The doctrine of animal welfare represents our cultural recognition that animals are not just things and that they matter morally. The doctrine of animal welfare is important to *our* moral identity. The doctrine of animal welfare helps make *us* feel better about continuing to exploit them. The problem is that, because animals are chattel property, the doctrine of animal welfare means very little to *them*.

Before the 19th century, at least in the West, animals were excluded altogether from the moral and legal community. There were, of course, some thinkers who rejected this and who thought that we had moral obligations that we owed to animals. But for the most part, it was, as a social, cultural, and legal matter, perfectly fine to ignore animal interests. Animals were regarded as inferior to humans. Although, in many instances, this inferiority was linked to theological notions in that only humans were deemed to be created in God’s image, the primary focus of this inferiority was cognitive. Animals supposedly lacked the sophisticated cognition of normally functioning humans; they lacked characteristics such as rationality, the ability to use abstract concepts, and self-awareness, and this was thought to justify our treating animals as things.

In the 19th century, a paradigm shift ostensibly occurred. We accepted that animal suffering was morally relevant, and that we had an obligation to treat animals “humanely” and to not impose “unnecessary” suffering on them. Although there were a number of reformers who contributed to formulating the animal welfare position, chief among them was the lawyer and philosopher Jeremy Bentham (1748–1832), who argued that the fact that animals lacked sophisticated cognition was irrelevant to whether animals mattered morally; all that mattered was whether animals could suffer. If they could suffer, then they had interests in not suffering and we could not justify excluding consideration of those interests when we were assessing actions any more than we could ignore the interests of slaves because of their race.

Bentham did not, however, maintain that we should stop using and killing animals. On the contrary, his view was that, although the cognitive differences between humans and nonhumans were irrelevant insofar as animal suffering was concerned, those cognitive differences were very relevant to the issue of killing. We could continue to use and kill nonhuman animals as long as we took animal suffering seriously because, unlike us, animals were not self-aware and had no interest in continuing to live. The cow did not care *that* we killed and ate her; she just cared about *how* we treated her and slaughtered her.

This thinking became what we now refer to as the animal welfare position. Animals, because they are sentient, have an interest in not suffering, but they do not have an interest in continuing to live because they are not self-aware. So we can use and kill them for our purposes as long as we treat them “humanely” and don’t impose “unnecessary suffering” on them. This gave us a green light to continue to exploit animals subject to the limitation that we accord appropriate weight to the interest of animals in not suffering.

The animal welfare position reflects our conventional wisdom when it comes to animal ethics. Indeed, the animal welfare position is so uncontroversial that it is included in laws that often impose a criminal sanction for violation. The problem is that this supposed limitation has proven to be no real limitation. As mentioned earlier, animals are property with an economic value. As I mentioned earlier, animals are property with an economic value and, because it costs money to protect animal interests, the level of protection we accord to their interests is generally low. Indeed, the treatment we accord to the animals who are the most “humanely” treated (the animals who are part of the niche market that goes beyond the legal requirements and supplies supposedly more “humane” animal products to those who are willing to pay more) are treated in ways that would be characterized as “torture” were humans involved. The killing of animals—however supposedly “humane” it is—requires that we buy into the nonsensical position that a sentient being does not have an interest in continuing to live because that being is not self-aware in the way that most humans are. We will examine this idea further in the answer to the fourth Question.

Animal welfare is not about animals; it is about making humans believe that we take animals seriously as a moral matter. Animal welfare is about making us feel more comfortable about continuing to exploit them. But animal welfare is a moral fantasy. The reality is that, because animals are property, they remain nothing more than things.

## Is It Acceptable to Eat Animals? If Yes, Why? If Not, Why Not?

No, it is not. I will present two arguments as to why it is not acceptable to eat animals. I will present the first argument in this section; I will present the second argument in addressing the fourth Question. However, I want first to make clear that when I talk about eating animals in this context, I am referring not only to meat, but to eggs, milk, and other food products made from animals. There is no morally coherent distinction between meat and any other animal products. They all involve suffering and death. As an ethical vegan, I also do not wear or otherwise use animal products or patronize circuses, zoos, etc. However, I will focus in this response on using animal products for food.

The first argument relies solely on the conventional moral principle that we all claim to accept—that it is morally wrong to inflict unnecessary suffering on animals. If “necessity” has any meaning in this context, it must mean that it is wrong to impose suffering on animals when there is no compulsion requiring that we do so. If we are imposing suffering for reasons of pleasure, amusement, or convenience, then we are, by definition, imposing suffering that is not necessary. The problem is that in an overwhelming number of cases, pleasure, amusement, or convenience are the *only* reasons that we are imposing suffering.

We kill approximately 80 billion land animals every year for food. They are all unquestionably sentient. We kill an unknown number of sea animals every year for food but a conservative estimate is one trillion. Even if some of these animals, such as clams and mussels, are not sentient, the vast majority of them are undisputedly sentient beings. Think about this: every year, we kill more sentient beings for food alone than the total number of humans who have lived on the earth through history—in other words, we are talking about a lot of animals. However, “humanely” these animals are treated (and as you know by now, I am very skeptical about the concept of “humane” exploitation), they all suffer. So that is a lot of suffering.

On what basis can we justify this? There are certainly instances in which humans choose to eat animals because, if they do not, they will starve to death. They have no other choice. How many humans are in this situation is debatable but, for present purposes, let’s just exclude them all. That leaves the rest of us—the vast majority—who *choose* to eat animal products because we like the taste or because it’s convenient or because it is just plain habit. *Everyone* reading this right now is in that category.

We don’t need to consume animal products for human health. Indeed, an increasing number of mainstream health professionals are maintaining that animal foods are detrimental to human health. Putting aside that humans are physiologically more like herbivores than carnivores, humans are omnivores and can get all the nutrition needed by consuming plants. What about B-12? Although some vitamin B-12 is made by bacteria in the human body, not enough is reliably made for our needs and the unhealthy habits that humans have prevent maximum production and absorption of the endogenous B-12. Therefore, it is necessary to supplement B-12 from external sources whether you consume a vegan diet *or* a diet of animal foods. *All* B-12 comes from bacteria—whether it is found in the gut of ruminating animals who get it from fermenting plant material in their hindgut, or from certain sorts of mushrooms or algae, water lentils, or supplements containing that bacteria. So if you adopt a vegan diet but don’t consume an alternative source of B-12, yes, you *may* get ill. However, there are plenty of people who have B-12 deficiencies, despite their consumption of animal foods.

How about DHA and EPA, the long-chain fatty acids that aren’t found in plant foods and that people eat fish to get? Most people can convert the short-chain fatty acids found in chia seeds, walnuts, dark leafy greens, and canola oil into long-chain fatty acids. Or you can get long-chain DHA and EPA directly from the source where fish get it—algae. There are now many vegan DHA/EPA supplements that are algae derived.

Given that we do not need to eat animals for reasons of health, how, then, can our consumption of animal products be considered as “necessary”? That’s easy; it can’t be. If we take seriously the conventional wisdom that we all claim to embrace and we believe that animals have some moral value, then, even without any consideration of animal rights, we cannot justify consuming animal products in any situation in which we have a meaningful choice.

So why aren’t those of us who are not stranded on a desert island or shipwrecked and drifting at sea, and who do have a choice (that’s most of us) not vegans at least as to our diet?

The answer goes back to the property problem. Because animals are property, we don’t ask whether animal use is necessary. We assume that animal use is acceptable as an exercise of human property rights even if the use is not necessary. When we apply our conventional moral thinking about animals to the matter of food, we ask only whether we are imposing gratuitous suffering in the production of meat, dairy, eggs, and other animal products. However, this is just an example of the general idea that I described earlier: because animals are property, thinking about animal welfare is generally limited to the level of protection that allows for economically efficient exploitation. So because animals are property, we do not ask whether the suffering of animals used for food is necessary; we ask whether it is necessary given a use that is not necessary. This is not anything that can be called a moral principle. It is nonsense but it’s convenient and it allows us to continue happily consuming animal foods.

In conclusion: if animals matter morally and are not just things that have no inherent or intrinsic value, then, even if we do not embrace an animal rights theory, we cannot justify eating animals or animal products in any situation in which we are not compelled to do so. When we choose to eat animal products, we are treating animals as things irrespective of what we otherwise say or believe.

## Do All Animals Deserve Equal Consideration in Terms of Animal Welfare?

I interpret this question as asking whether our obligations to animals should vary according with the various capacities and cognitive characteristics of different species; that is, do we have different obligations toward nonhuman great apes than we do chickens? As I have tried to make clear in my answers to the previous Questions, it is my view that the primary obligation imposed by the animal welfare standard is to exploit animals in an efficient way. We can use them for all sorts of unnecessary purposes but we should not impose any more suffering on them than that which is required to use them for these (almost always) unnecessary purposes. I will instead argue that the principle of equal consideration requires that we treat all sentient beings equally in one respect: we should use none of them exclusively as a means to our ends. We should accord all sentient beings—irrespective of their other characteristics—a right not to be used exclusively as resources.

As I discussed in response to the third Question, it is not—even according to any rational interpretation of our conventional thinking about animal ethics—morally acceptable to eat or otherwise use and kill animals in the absence of a compulsion. I will now offer another reason why the answer to the third Question must be “no” that rests on the idea that all sentient beings are equal in at least one respect. As part of this, I will argue that the distinction that is the basis of the animal welfare position between having an interest in not suffering and having an interest in continuing to live is nonsensical and anthropocentric.

The primary characteristic of personhood is having a morally significant interest in continuing to live. For example, when we talk about whether a human fetus is a person, we are not asking whether a human fetus is human; we are asking whether the fetus has a morally significant interest in continuing to live. We generally associate human personhood with the characteristics of moral agency—that is, the cognitive characteristics that are required to make moral decisions. But how do we deal with humans who lack these characteristics? For example, humans who are experiencing late-stage dementia are stuck in an eternal present. They may enjoy every second of their lives but have no connection with a future self beyond the next second of their consciousness. They have no awareness of self beyond their awareness of self in that second, the next second, and so on. Is such a human “equal” to a normally functioning human? It depends on the context of the question.

If the question is whether these two humans are equal for the purpose of deciding whom should be appointed as a history lecturer in a school, the answer is that the two are not equal. However, if the question is whether the two are equal for purposes of deciding whom we should use as a forced organ donor or as a nonconsenting subject in a biomedical experiment that will not cause pain but will end in death, the answer is—at least for most of us—that we should use neither in this way. We think that both have a moral significant interest in their lives.

Why do we think that? I believe the answer is clear. Where humans are concerned, we recognize that any sentient human—any human who is subjectively aware—has an interest in continuing to live even in the absence of the cognitive characteristics that we associate with normally functioning human agents. We recognize that, in the human context, we cannot separate an interest in not suffering from an interest in continuing to live. If we were to say, as Bentham did concerning nonhuman animals, that a human with late stage dementia does not care about whether we kill him but only that we not inflict any pain in the process, we would overlook the fact that killing such a human deprives him of the entirety of his consciousness—something he values however it is he values it. This is why we think that all humans who are subjectively aware should enjoy a right to life—that is, that their interest in continuing to live, their desire to get to the next second of their consciousness, should be protected even if the consequences of ignoring that interest would be beneficial for the rest of us. A right is simply a non-consequential way of protecting an interest.

If we take a step back and consider this separation of sentience from an interest in continuing to live in a human context, we see how absurd it is. If I were to say that a human with dementia was indifferent to whether his consciousness ended but only cared about not experiencing pain in the process, you would rightly point out that such a human values his life *however* he values it and that we cannot morally justify ending that consciousness particularly if the human is otherwise not in constant pain (and even then, we think that killing such a person raises serious moral issues).

I would like to suggest that Bentham got it wrong. He maintained that sentience was the only characteristic necessary for moral significance, but that more was required in order to have a morally significant interest in continuing to live. I understand the motivation: Bentham wanted to continue to eat animals, so such a distinction was required. But that does not mean that the distinction is sound. It isn’t.

Although we may disagree about whether certain conduct involves discrimination, we are all agreed that chattel slavery is morally odious because it involves treating a human as property and allows someone else—an owner—to value the fundamental interests of that human and this results in the enslaved human being excluded altogether from the moral and legal community. We maintain that whatever other rights a human must have in order to be a person, the human must at least have a right not to be the property of others. The same analysis applies to nonhumans. If animals are going to be members of the moral community, they cannot be property. Bentham realized that human slavery was problematic in that it systematically devalued the interests of slaves relative to the interests of owners. The same problem occurs when nonhumans are property.

My views here are explored at greater length in these works:

*Why Veganism Matters: The Moral Value of Animals* (Columbia University Press, 2020); *Eat Like You Care: An Examination of the Morality of Eating Animals* (Exempla Press, 2013) (with Anna Charlton); *The Animal Rights Debate: Abolition or Regulation?* (Columbia University Press, 2010) (with Robert Garner); *Animals as Persons: Essays on the Abolition of Animal Exploitation* (Columbia University Press, 2008); *Introduction to Animal Rights: Your Child or the Dog?* (Temple University Press, 2000); *Rain Without Thunder: The Ideology of the Animal Rights Movement* (Temple University Press, 1996); *Animals, Property, and the Law* (Temple University Press, 1995).

## About the Author

Gary L. Francione is Board of Governors Distinguished Professor of Law and Nicholas deB. Katzenbach Scholar of Law and Philosophy at Rutgers University Law School, Visiting Professor of Philosophy at the University of Lincoln (UK), and Honorary Professor of Philosophy at the University of East Anglia (UK). His most recent book is Why Veganism Matters: The Moral Value of Animals.

